# Dynamic electronic institutions in agent oriented cloud robotic systems

**DOI:** 10.1186/s40064-015-0810-4

**Published:** 2015-03-01

**Authors:** Vineet Nagrath, Olivier Morel, Aamir Malik, Naufal Saad, Fabrice Meriaudeau

**Affiliations:** Laboratoire Le2i, UMR CNRS 6306, Le Creusot, 71200 France; Universiti Teknologi PETRONAS, 31750 Tronoh, Perak, Malaysia

**Keywords:** Dynamic electronic institutions, Cloud robotics, Model driven engineering, Cloud computing, Peer-to-peer system, Business model

## Abstract

The dot-com bubble bursted in the year 2000 followed by a swift movement towards resource virtualization and cloud computing business model. Cloud computing emerged not as new form of computing or network technology but a mere remoulding of existing technologies to suit a new business model. Cloud robotics is understood as adaptation of cloud computing ideas for robotic applications. Current efforts in cloud robotics stress upon developing robots that utilize computing and service infrastructure of the cloud, without debating on the underlying business model. HTM5 is an OMG’s MDA based Meta-model for agent oriented development of cloud robotic systems. The trade-view of HTM5 promotes peer-to-peer trade amongst software agents. HTM5 agents represent various cloud entities and implement their business logic on cloud interactions. Trade in a peer-to-peer cloud robotic system is based on relationships and contracts amongst several agent subsets. Electronic Institutions are associations of heterogeneous intelligent agents which interact with each other following predefined norms. In Dynamic Electronic Institutions, the process of formation, reformation and dissolution of institutions is automated leading to run time adaptations in groups of agents. DEIs in agent oriented cloud robotic ecosystems bring order and group intellect. This article presents DEI implementations through HTM5 methodology.

## Introduction

### A note to practitioners

Cloud computing is a business model for the internet. A typical scenario of cloud computing has a serving party that offers its infrastructure, platform or software resources to one or many clients across the network cloud. Cloud service businesses charge their clients based on the quality and volume parameters chosen as and when required by the client. Service contracts, banking and administrative mechanism created the trust envelop that made cloud computing business model a success. When we move the ideas of cloud computing to robotics, there are two kinds of adaptations that will take place. The first kind of adaptation will involve direct modification of cloud services to suit robotic applications while the second kind of adaptation will be on the lines of social and business ideas represented by cloud computing. We believe that this second kind of adaptation will require special tools and development methodologies. Cloud robotic entities include all robotic and non-robotic entities that collectively build a cloud robotic service ecosystem. Using software agents to represent cloud robotic entities will require minimal changes in the way those entities are independently developed by various vendors. Agents are also ideal for implementing social and business concerns of a cloud robotic entity. HTM5 (5 View Hyperactive Transaction Meta Model) is a 5-view meta-model for model driven development of agent oriented cloud robotic systems. The trade view of HTM5 promotes peer-to-peer exchange of services based on relationships and contracts between participating agents. Agents are autonomous entities with personal goals that may make them greedy in their interactions with other agents. Dynamic Electronic Institutions are modelled on the ideas of Institutions in Human societies. Norms based on trade contracts, social relationships and institutions bring a sense of order in multi agent systems. The aim of this article is to test feasibility of HTM5 methodology in implementing Dynamic Electronic Institutions.

### Background

Cloud computing is a relatively new business model for the Internet. NIST (National Institute of Standards and Technology- United States) defines cloud computing as *"a pay-per-use model for enabling available, convenient, on-demand network access to a shared pool of configurable computing resources that can be rapidly provisioned and released with minimal management effort or service provider interaction."* Cloud computing does not introduces a new computing or network technology but as a business model it remoulds the way existing technologies are used. Decreasing cost of internet connectivity and cheaper internet enabled devices has further improved the feasibility of cloud computing as a business model. Robotic researchers and engineers soon realized the benefits of cloud computing in robotics. Cloud based storage and processing expanded functionalities while carrying a minimal set of hardware on-board. Emergence of cloud robotics from cloud computing can be seen as a twofold development. The more visible development is direct modification of current cloud based services for robotic applications. Cloud robotics is a comprehensive term used to describe infrastructure, platform or software as a service for robots, internet enabled robotics, utilisation of search engines by robots and use of internet for communication between robots. These developments are making an impact in the way robotic systems are designed using cloud based tools but not much is done towards developing cloud robotics as a business model.

**Figure 1:**
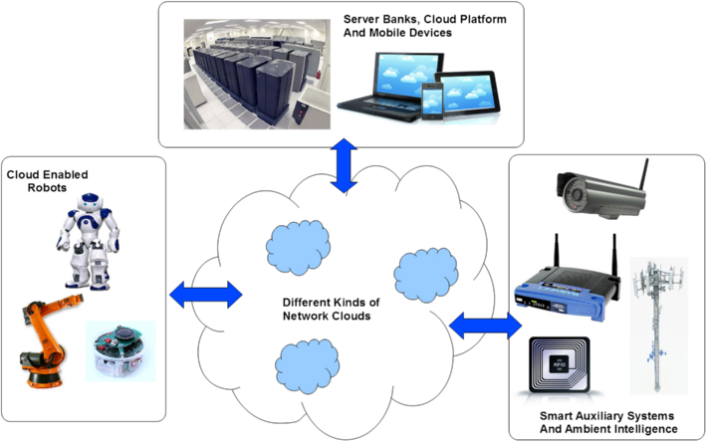
**Robotic, non-robotic and network elements of a typical cloud robotic ecosystem.**

Development of cloud robotics as a business model will require new tools and methodologies. It is essential to develop methodologies that are industry and business oriented. The cloud robotic methodologies should go one step further to include models that incorporate concepts like Distributed Artificial Intelligence (DAI) (Stone and Veloso 2000), registry based service discovery and automated matchmaking mechanism. Many of the services offered by a robot to other robots will have a physical world component. A robot’s physical reach will determine the scope of the physical services offered by it and any business model for a robotic ecosystem should include provisions to model factors that codify physical world interactions. A cloud robotic ecosystem will also include many non-robotic entities. These entities could range from ambient intelligence to server banks. In theory any device that can communicate through a network could be included as a working component of a cloud robotic system. The communication networks that collectively build the cloud could be of different kinds and visible in selective physical regions. A methodology that allows modelling of these non-robotic devices, networks and interfaces will give a complete design toolset to designers of cloud robotic systems. Figure 1 shows a typical cloud robotic ecosystem with robotic and non-robotic entities. A design methodology for cloud robotic ecosystem should provide tools to model all physical and theoretical aspects of these systems. Key theoretical elements of a cloud robotic ecosystem would be its network structure, event driven behaviour, social interactions, norm driven peer-to-peer trade, micro level competitions and dynamically regulating collaboration (Weiss 1999). A usable agent oriented cloud robotic methodology could concentrate only on the implementational design aspects and system requirement capture. The reason why a metamodel with tools to include DAI is useful is because as the systems become more robust and extensive, it will require mechanism to implement some level of intelligence. Theoretically such intelligence can be implemented at the running code level irrespective of the methodology used to envision the system. The decision to include DAI friendly elements in all three layers of the design (computation independent, platform independent and platform specific layers) makes the system more suitable for researchers as well as engineers who will develop the DAI concepts on agent oriented cloud robotic system.

An agent oriented approach towards cloud robotics has some distinct advantages. A typical cloud robotic entity will have a manufacturer with a personalized development methodology for its product. The manufacturer would typically like to hide the internal designs of its product and thus the business that deploys that entity may have limited or no access to the internal software framework of the entity. The business will also want to keep its business logic hidden from the outside world and even from sections of their own workforce. “*Software Agents are computational entities with specific roles and personal objectives working in a visible environment with other entities which may have dissimilar roles and objectives*” (Jennings and Bussmann 2003). Using a software agent to represent cloud entities does not interfere with manufacturer’s development methodology. Agents are closed autonomous systems that have an internal logic framework that communicates with the outside world via messages. Unlike objects in an object oriented methodology, Agents do not release details of their functionalities and do not allow direct execution of their functions by other agents. Agents thus are by design ideal for implementing a secure business logic. Multi-agent systems (Luck et al. 2003, 2005; Wooldridge 2002) are also ideal for implementing intelligent concepts like Distributed Artificial Intelligence (DAI) (Stone and Veloso 2000) and digital business ecosystem (Discussed in Section 2, 2). An agent based approach is idea for systems with dynamic participation of entities in an open (Hungate and Gray 1995) peer-to-peer service exchange.

**Figure 2:**
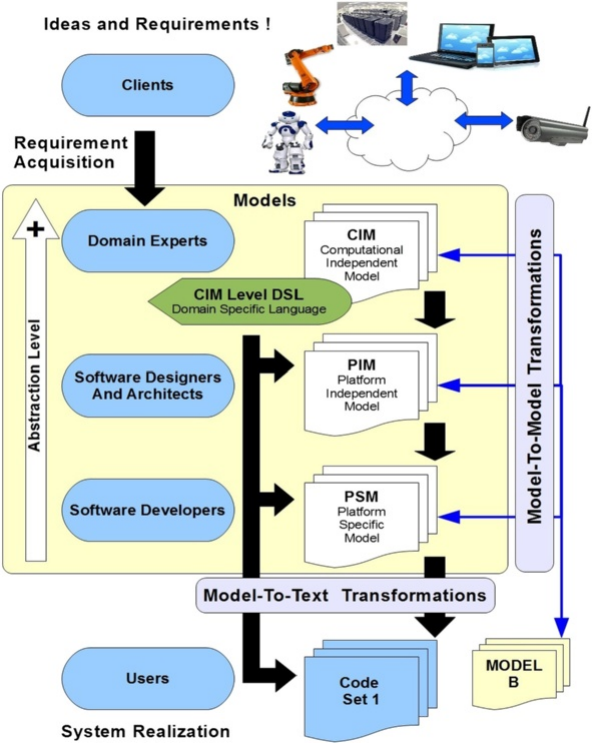
**Object management group’s model driven architecture (OMG-MDA) (** OMG 2003 **).**

Object Management Group’s Model Driven Architecture (OMG-MDA) (OMG 2003) is a prevalent industrial standard for development of software Meta-models for complicated systems. A model is a set of valid comments about a system and a Meta-model is a set of valid comments about a model (Seidewitz 2003). Model Driven Engineering (MDE) develops system models with high level of abstraction, without much emphasis on implementational details. MDE is ideal for Systems where the overall idea of a system (and not its implementation) is more important at initial stages of development. More than one models for the same system may be made to separate system concerns. These multiple models are called views of the system and the practice is known as multi-view methodology (Finkelstein et al. 1992). Software Product Line Engineering (SPLE) (Clements and Northrop 2001; Pohl et al. 2005; Weiss and Lai 1999) is an encouraged practice in software industry to produce reusable system components and models. Methodologies which comply with OMG-MDA and SPLE standards have higher industrial acceptance. OMG-MDA has a three layer architecture where layers vary in their level of abstraction and target audience. Figure 2 shows the three layers of a typical OMG-MDA meta-model with Computation Independent (CIM), Platform Independent (PIM) and Platform Specific (PSM) models at different layers. Computation Independent, Platform Independent and Platform Specific layers of OMG-MDA cater to different stakeholders in the development life cycle. Computation Independent or Platform Independent models may be supported by a Domain Specific language of same layer abstraction. The Domain Specific Language can be executed to generate automated Model to Model and Model To Text (Code) transformations. A Domain Specific Language (DSL) is often built to support a Meta-model. Code written in a DSL is used to codify designs in a programming language that has a domain specific syntax based on a particular Meta-model. DSL code can be made executable by writing compiler functions to generate Model to Model and Model to Text transformations. These transformations are essential as they allow a new Meta-model to be translated into existing Models/Languages.

**Figure 3:**
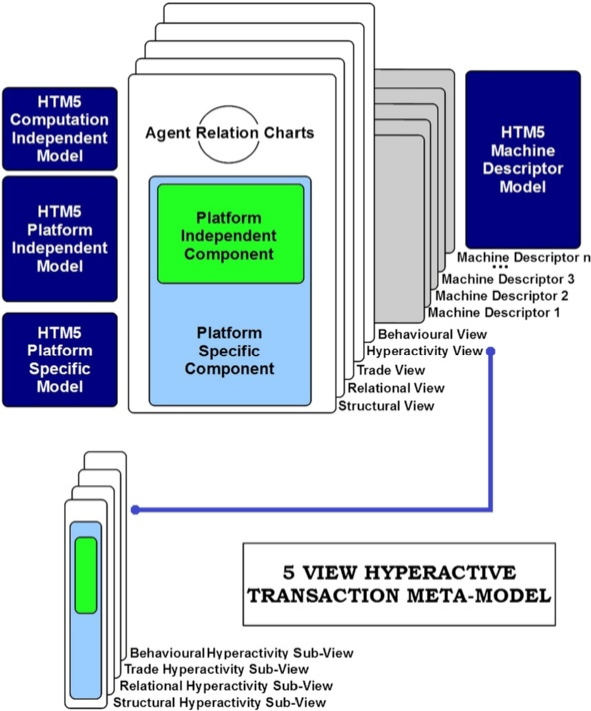
**An overview of 5 views hyperactive transaction meta model (HTM5) for agent oriented development of cloud robotic systems.**

The 5-view Hyperactive Transaction Meta-Model (HTM5) is a domain specific Meta-model for agent oriented development of cloud robotic systems. HTM5 is based on OMG-MDA and has the prescribed three layer architecture as shown in Figure 2. The 5 views and 4 hyperactivity sub-views in HTM5 (Figure 3) separate view-specific concerns in all three layers. The Platform independent and platform specific layers of HTM5 are component level layers and are developed in two phases. The first phase creates class component templates for individual agent components which are then developed by various entity manufacturers in second phase of development. HTM5 also has a Machine Descriptor Model (HTM5-MDM) that models machine (host hardware or software entity) represented by an agent component. Topmost layer in HTM5 has a set of Computation Independent graphical models named Agent Relation Charts (ARCs). The term Hyperactivity in HTM5 corresponds to provisions that allow deviations from ideal Agency as and when required. Hyperactivity may be used to give certain Agents, an object like characteristics by releasing their autonomy for specific associated Agents. HTM5 follows multi-view meta-modelling methodology and has 5 separate views to capture structural, relational, trade, hyperactivity and behavioural elements of the design. Some components of HTM5 Meta-model were introduced in earlier publications (Nagrath et al.2013a,2013b). The trade-view of HTM5 promotes peer-to-peer trade amongst software agents. HTM5 agents represent various cloud entities and implement their business logic on cloud interactions. Trade in a peer-to-peer cloud robotic system is based on relationships and contracts amongst several agent subsets. Dynamic Electronic Institutions (Section 2) are modelled on the ideas of Institutions in Human societies. Norms based on trade contracts, social relationships and institutions bring a sense of order in multi agent systems. The aim of this article is to test feasibility of HTM5 methodology in implementing Dynamic Electronic Institutions. For the benefit of the reader, we will elaborate aspects of HTM5 relevant to the subject matter of the current article.

A view by definition is a representation of the system with concerns specific to a particular stakeholder. In a multi view model it is essential that the designer should have a clear idea about the scope of a particular view. Trade view of HTM5 is for modeling the trade logic in a cloud robotic ecosystem. HTM5 proposes a Peer-to-peer service oriented trade mechanism managed by *Relation* agents. For a system designer, it is important to specify the norms and default trade relationships in a cloud robotic system. Agent transactions in cloud robotic systems follow the cloud computing business logic, but unlike current cloud robotic systems, these transactions are driven by decentralized *Relation* agents. The Trade view of HTM5 targets to capture the following design elements of an agent oriented cloud robotic ecosystem:

1. **Names** and **Relative Locations** of the following *Trade* elements in a cloud robotic ecosystem.
  - Components (*Agent, Relation* or *Merge*) that are involved in Trade.
  - Items in Trade.
  - Data entities associated with Trade items.
2. The following **Information** about the above entities:
  - Associations between Trade items and Components.
  - Nature of association between a Trade item and a Component:
    (i) Item is a Demand by a Component.
    (ii) Item is a Service provided by a Component.
  - Entities associated with a Trade item:
    (i) Components that provide the item as a service.
    (ii) Components which demand the item.
    (iii) Data entities which are associated with Trade of the item.
    (iv) The Components (Generally *Relations*) that are hosting and managing those data entities.
  - Nature of various Data entities:
    (i) Is it a Lookup table?
    (ii) Is it a cost metric?
    (iii) Is it a management variable?
3. Following **Functionalities** should exclusively go in the Relational view classes of various components:
  - Localization: Locating one’s position in different transactions.
  - Identifying relationships associated with a particular trade item.
  - Implementing relationship norms associated with a trade item.
  - Implementation of Business logic of a Component (Functionality related to business concerns of a particular HTM5 Component).
  - Implementation of Business logic of the system (Functionality related to business concerns of the cloud robotic system).
  - Calculating readjustments in relationship norms based on business logic.
  - Communicating desired readjustments to relational view classes.
  - Maintaining data entities associated with a trade item.
  - Reading and updating of remotely hosted data entities associated with a trade item.
  - Generating triggers for Trade Hyperactivity sub-view class. (Initiation, management or finalization of a Hyperactive link).

## Dynamic electronic institutions

Human societies are amalgamation of several norm based institutions that give order to otherwise random interactions. Institutions are structures based on mutual incentives based on predefined contracts. Social, Political and economic institutions represents the norms of a society and interactions of its members. Institutes establish standardization in response from a member entity which in absence of an institution is free to act solely for its own benefit. Institution helps in controlling the greediness of individual entities and brings order to a system (North 1996). Electronic Institutions are a relatively new field where the concept of human institutions is extended to Multi Agent Systems (MAS) (Luck et al.^2003^,^2005^; Wooldridge^2002^) and Distributed Artificial Intelligence (DAI) (Stone and Veloso 2000). Some early attempts towards the use of organizational metaphors for system modelling systems were presented in (Pattison et al.^1987^; Werner^1989^). The first approach towards electronic institutions was given in (Noriega 1999) where an abstract notion *agent-mediated electronic institution* was introduced for the first time. These institutions are described as environments where agents are interacting with other agents under predefined restrictions. An institution is specified by a set of pre-defined norms that restrict actions of its member agents. The idea of an electronic institution is very open and various groups (Aldewereld et al.^2005^; Dignum^2004^; López 2003) are working on this problem with different perspectives. Electronic institutions require limited human intervention for institution design phase. In open agent systems, it is necessary to automate the design phase of institutions.

**Figure 4:**
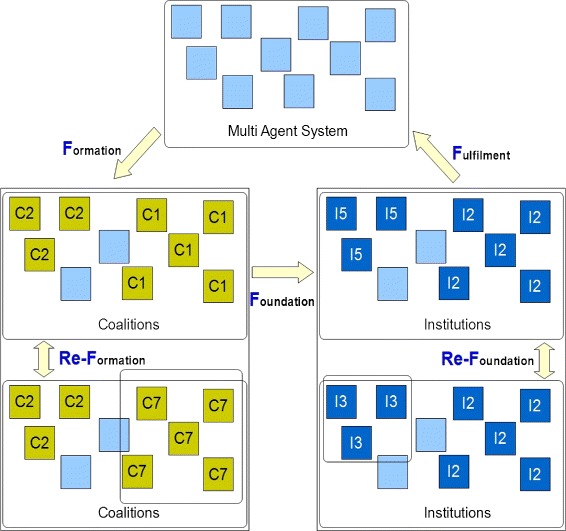
**3F life cycle of a dynamic electronic institution.** The three phases are in order: Formation, Foundation and Fulfilment. Re-Formation and Re-Foundation processes are also within the 3F life cycle.
- Re-Formation: Re-formation is the process of reconfiguring a coalition. A reformation may be triggered by change in coalition membership or a change in parameters responsible for coalition formation.
- Foundation: The member agents in a coalition choose a candidate Institution to form. The norms of the candidate institution are based on collective views of individual members of the coalition. Once the target institution is selected, the coalition goes through institutionalization to form an institute of the selected type. This step presents a lot of challenges as selection of the kind of institution and maintenance of institution-base requires a well-defined strategy. In theory, any strategy that assigns an institute to a group of agents (based on their collective decision parameters) qualifies as a *Foundation Logic*.
- Re-Foundation: Re-foundation is the process of reconfiguring an institution. A reconfiguration may be triggered by a change in environment variables or a foundation-timeout value set by *Foundation logic* (at the time of institution foundation).
- Fulfilment: The member agents in an institute dissolve into individual free agents when the institute completes all its objectives. An institute may also fulfil when triggered by a fulfilment-timeout value set by *Foundation logic* (at the time of institution foundation). Like foundation, fulfilment is also a challenging logic to device. The decision may be based on weighted percentage of collective goals of member agents, or time elapsed since institution foundation / re-foundation. *Fulfilment logic* is usually devised at foundation state when the institute candidate is selected by *Foundation logic*.

The term *Dynamic Electronic Institutions* first appeared in the roadmap for agent technology (Luck et al. 2003). Dynamic Electronic Institutions are Electronic Institutions where formation, reformation and dissolution of institutions are automated processes. The norms and objectives of institutionalization are dynamically adapting to the needs of its member agents. Figure 4 shows the 3-F life cycle of an institution proposed in recent works (Muntaner-Perich and Rosa Esteva 2007;^2008^). Re-Formation and Re-Foundation processes are also incorporated in the 3-F life cycle.

- Formation: Agents with similar objectives come together to form a coalition. A coalition is usually not governed by a set of norms, but trust between agents may play a part in the coalition formation phase. Any logic that governs coalition formation between a set of agents is their *Formation logic*.

## Dynamic electronic institutions in cloud robotics

There are many application domains where Dynamic Electronic Institutions (DEIs) are applicable. In agent oriented cloud robotics, DEIs are applicable in tasks involving a contract based cooperation amongst ad-hoc teams. Collaboration between agents which were not originally programmed to work together is examples of Ad-Hoc teams. Common scenarios where DEIs are used are Ad-Hoc mobile networks, B2B (Business to Business) electronic commerce and OOTW (operations other than war) scenario. For the current article we will concentrate on B2B electronic commerce as a scenario for agent oriented cloud robotic system. Following the 3F life cycle in B2B electronic commerce, following analogy was proposed by (Muntaner-Perich and Rosa Esteva^2007^,^2008^).

- A Digital B2B electronic commerce ecosystem: *Agent Community*
- A Business Opportunity: *Coalition*
- Digital Business Ecosystem (DBE): *Dynamic Institution*

**Figure 5:**
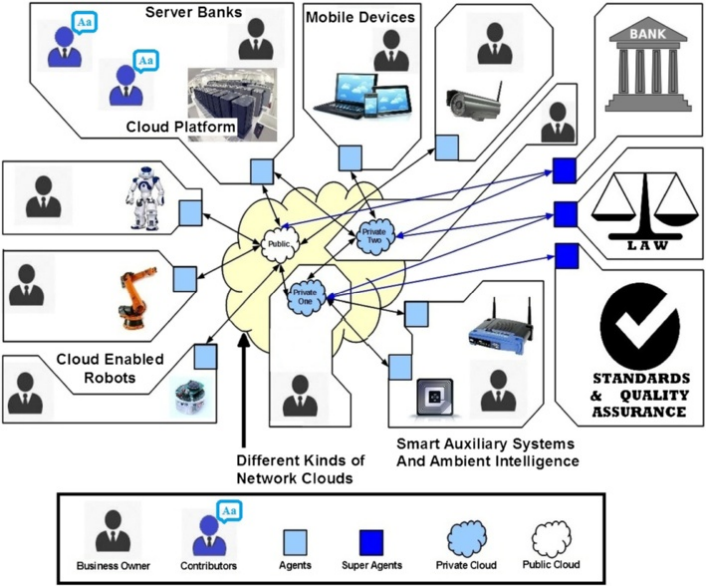
**Example of digital business ecosystem (DBE) in an agent oriented cloud robotics environment.**

The aim of the DEI scenario mentioned above is to find new opportunities to exchange services amongst member agents. Formation of a coalition represents a viable service exchange (a business opportunity). When the member agents agree upon a set of norms to execute the service exchange, it is represented as foundation of an institution. Figure 5 shows an example where DBE is applied to an agent oriented cloud robotic ecosystem. Agents are independent software entities that represent a robotic/non-robotic entity in cloud robotic ecosystem. Individual cloud entities may have different hardware and software setup. Each of these entities may have a business owner and set of offered services. Some online server banks may have a number of contributors which build up a resource (like algorithms and other internet resources) which is then offered as a service to other entities through cloud. An open registry and matchmaking service allows peer-to-peer trade of these services in cloud robotic ecosystem. The network cloud itself may have many private and public networks, some of which may be owned by businesses that allows their use as a service. Banking super agents (Agents with special access norms) allow actual transfer of money between agents. Other super agents may be present to enforce law, quality, communication and trade standard over the agent community. Development life cycle of cloud robotic products may differ from vendor to vendor. Business model of organisations that deploy these products may also differ. Individual entities may enter or exit the ecosystem dynamically and their behaviour may change with time. An approach based on representative agents ensures that heterogeneity in business logic, design methodology and implementation of these products is respected. An approach based on representative agents ensures that heterogeneity in business logic, design methodology and implementation of these products is respected. In a real life scenario, it may be required to have a minimal human involvement in the 3F life cycle (see Figure 4). A proposal by (Muntaner-Perich and Rosa Esteva 2007;^2008^) advocates a 7 step life cycle where human decision makers are involved at two stages to validate business opportunities detected by the DBE system.

Steps of the DBE lifecycle (Muntaner-Perich and Rosa Esteva 2007;^2008^):

1. Search of opportunities (Formation Logic)
2. Analysis of opportunity by business owner (Validation I, Optional)
3. Coalition establishment (Formation and Re-Formation Phase, Re-formation will require re-validation)
4. DBE selection (Foundation Logic)
5. Acceptation by business owner (Validation II, Optional)
6. DBE establishment (Foundation and Re-Foundation Phase, Re-foundation will require re-validation)
7. DBE Finalization (Fulfilment Phase)

## Dynamic electronic institutions in HTM5

In the previous section we saw the steps in a DBE lifecycle and its applicability in the cloud robotic domain. HTM5 (Nagrath et al.2013a,2013b) is OMG-MDA (OMG 2003) based meta-model for development of agent oriented peer-to-peer cloud robotic systems (See Section 2). The meta-model is designed to provide tools to implement advance distributed Artificial Intelligence (DAI) designs in a cloud robotic ecosystem. In this section we discuss the tools and anatomical elements of HTM5 that are utilized to implement a Digital Business Ecosystem based on Dynamic Electronic Institution.

**Figure 6:**
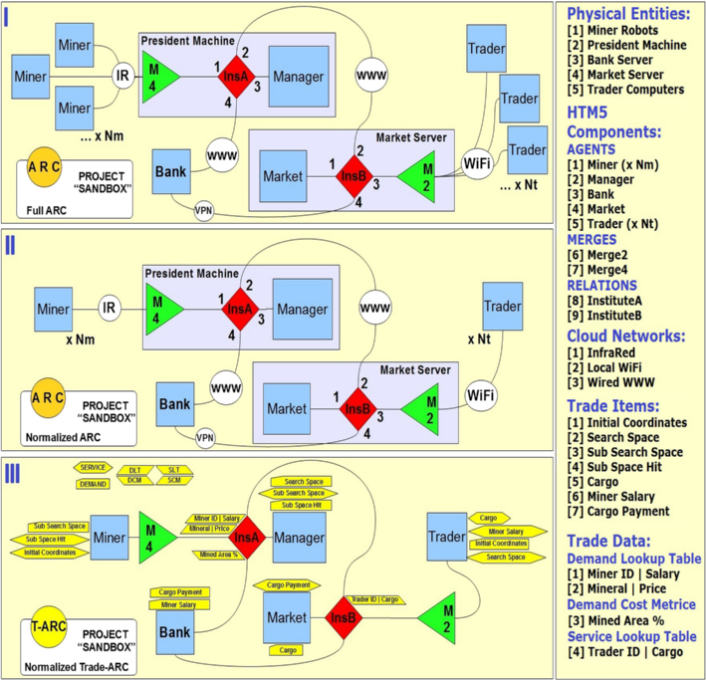
**An example of dynamic electronic institutions implemented using HTM5 methodology.** Above are agent relation charts (Computation independent layer of HTM5) for structural, relational and trade views of HTM5.

Figure 6 shows an example of a Dynamic Electronic Institution with two institutions. The HTM5 methodology allows for special agents called *Relations* that maintain the relationships between groups of agents in a relationship. The sample project *Sandbox* is a Digital Business Ecosystem example with two institution seeds. Institutes (Relation Agents) manage trade within an institute and host service and cost lookup tables along with other trade data items. Social and business logic of an Institute is implemented at relation agents. Detailed description of ARCs and HTM5 anatomy can be found at (Nagrath et al.^2012^,2013a,2013b). The above example locates elements of Digital Business Ecosystem in a HTM5 based implementation. Section 2 of this article presents a complete case study of DBE implemented using HTM5 methodology.

For the example shown in Figure 6, the relations are designed to act as Institution seeds. No anatomical change is required in HTM5 to use *HTM5 relation* construct as Institution seeds. An institution between groups of agents can be seen as a special kind of relationship. In HTM5, the norms and relationship variables of a relationship are hosted and managed by the *Relation* agent. When HTM5 is used to implement Dynamic Electronic Institutions, the variables, formation and foundation logic may be hosted in institution seeds (which are relation agents). For ease of implementation, an institution may be implemented as two separate agents. In Figure 6, the *Manager agent* along with *InsA relation* and *M4 merge* are all hosted at one machine. It is possible to implement the logic of all three components (Manager, InsA and M4) onto one agent but separation and placement of machine functionalities into agent, merge and relation specific parts is encouraged in HTM5. In Figure 6 Part I of the figure is a full ARC diagram of the *Sandbox* system while part II is its normalized version. There is multiple numbers of Miner and Trader agents in the system. Part III models trade dependencies in *Sandbox* cloud ecosystem. Peer-to-Peer trade relationships exists between members of a relationship (Here relationships are modelled as Institutions). The Trade-Agent Relation Chart (Trade-ARC) that defines the following trade relationships and dependencies between members of *Sandbox* cloud robotic ecosystem.

1. Trade **Search Space** is a service provided by *Trader* to *Manager* and assigns a search space in the mine for the *Manager*.
2. Trade **Sub Search Space** is a service provided by *Manager* to *Miners* and assigns a section of the search space (allocated to the *Manager*) to the *Miner*.
3. Trade **Sub Space Hit** is the notification service from the *Miner* agent when it detects the target mineral. This service is a demand at *Manager* agent.
4. Trade **Initial coordinates** locates the found mineral in the mine field. This service is a demand at *Trader* agent.
5. Trade **Miner Salary** is the payment that a *Miner* agent receives in exchange of the mineral locations it delivers to the *Trader* agent. The service is a demand at the *Bank* agent which transfers the amount from *Trader’s* to *Miner’s* account.
6. Trade **Cargo** is a service by the *Trader* agent to the *Market* where it sells the acquired mineral locations.
7. Trade **Cargo Payment** is the payment that a *Trader* agent receives in exchange of the mineral locations (Cargo) it delivers to the *Market* agent. The service is a demand at the *Bank* agent which transfers the amount from *Market’s* to *Trader’s* account.
8. Demand Lookup Table **Miner ID | Salary** is a lookup table to get the desired salaries (Trade: **Miner Salary**) by individual *Miner* agents.
9. Demand Lookup Table **Mineral | Price** is a lookup table to get the prices of different minerals (Trade: **Sub Space Hit**) by individual *Trader* agents. A *Miner* agent chooses its target mineral based on the current price of minerals.
10. Demand Cost Metrice **Mined Area Pc.** Is a trade variable to check the percentage of mine’s area that is already explored. This is a metrice to know when to allocate a new search space (Trade: **Sub-Search Space**) to individual *Miners*.
11. Service Lookup Table **Trader ID | Cargo** is for the trade **Cargo Payment** and is used by the *Market* agent to initiate cargo payments.
12. Institution **InsA** is an institution between **one***Manager* agent, **one** relation *InsB*, **one***Bank* agent and **Nm***Miner* agents. The institution manages the allocation, reallocation and management of mine spaces for individual *Miners* and allows for dynamic updating of a *Miner’s* asking salary based on inputs from *Bank* (Miner’s current bank balance) and *Market Server* (updated prices of different minerals).
13. Institution **InsB** is an institution between **one***Market* agent, **one** relation *InsA*, **one***Bank* agent and **Nt***Trader* agents. The institution manages the allocation, reallocation and management of Cargo items for individual *Traders* and allows for dynamic updating of a *Trader’s* asking price for individual minerals based on inputs from *Bank* (Trader’s current bank balance) and *President Machine* (updated percentage of Mined area).

## Case study

**Figure 7:**
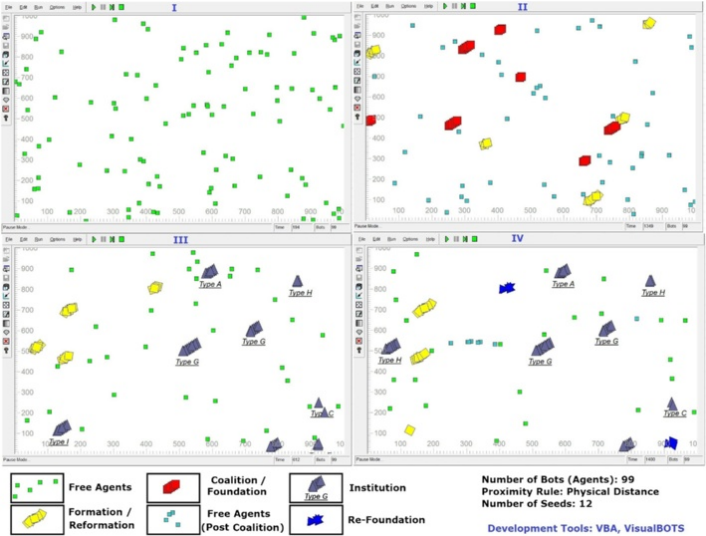
**Above are some instants from dynamic electronic institutions based digital business ecosystem simulations.**

The case study for the use of HTM5 methodology in developing a Dynamic Electronic Institution based Digital Business Ecosystem was executed in two phase. In the first phase computer simulations using VisuaBOT (VisualBots Simulator 2014) and VBA (Visual Basic for Applications 2014) were developed for conducting various experiments on the agent colony. Economic comparisons made between agent colonies working with and without dynamic electronic institutions. In the second phase, a scaled down version of the experiments performed on the simulations were implemented on five physical robots (TurtleBOTs). The principle motivation for these case study experiments is to show HTM5’s feasibility in implementation of a Dynamic Electronic Institution based cloud robotic ecosystem. This section discusses the system design, experiments, results and key observations. HTM5 is proved to be a usable methodology for this implementation since the experiments were performed using anatomical and design constructs prescribed by HTM5. Figure 7 show some instances from the simulated experiments. Figure 7 Part I shows a colony of BOTs (Agents) randomly moving in an environment. There are seed BOTs which are not moving and they act as the open ended joining point for other BOTs to form a coalition or an institution. Figure 7 Part II shows some of the seeds in Formation phase (3F life cycle) while some of the groups have formed a coalition and are now in Foundation phase. Figure 7 Part III shows formation of eight Institutions of different cardinality and type. Some of the groups are in Formation or Re-Formation phase. In going from Part III to Part IV two institutions have moved to Re-Foundation phase while one of the institutes (of Type I in Figure 7 Part III) has finalized freeing its member BOTs. All simulations are run with different experimental conditions and against different Market trends. When institutions are not allowed to be formed, the groups come together without setting up a set of norms and the dissolve as soon as a minimal number of member BOTs are unhappy with the current market direction/trend.

**Figure 8:**
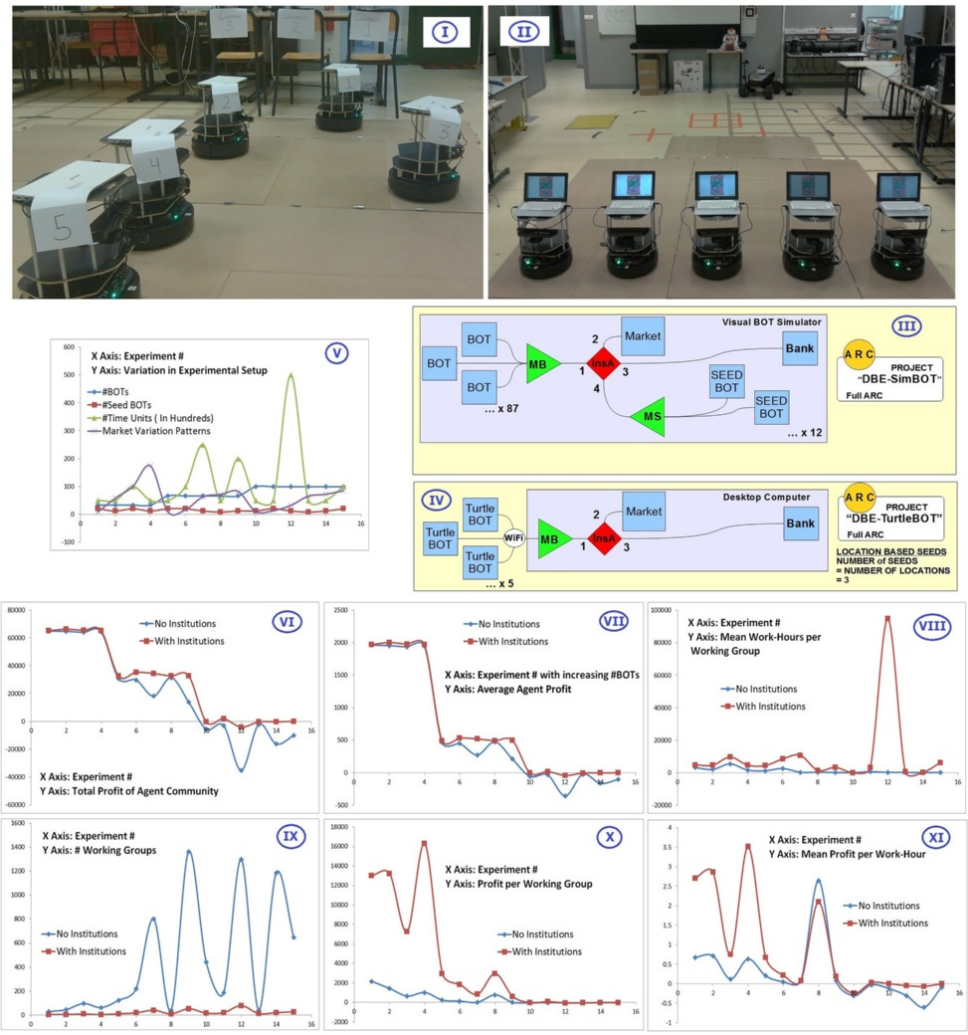
**ARC designs, physical robot experiments, simulation variability and results.** The key observations from these representations are explained in Section 2.

**Figure 9:**
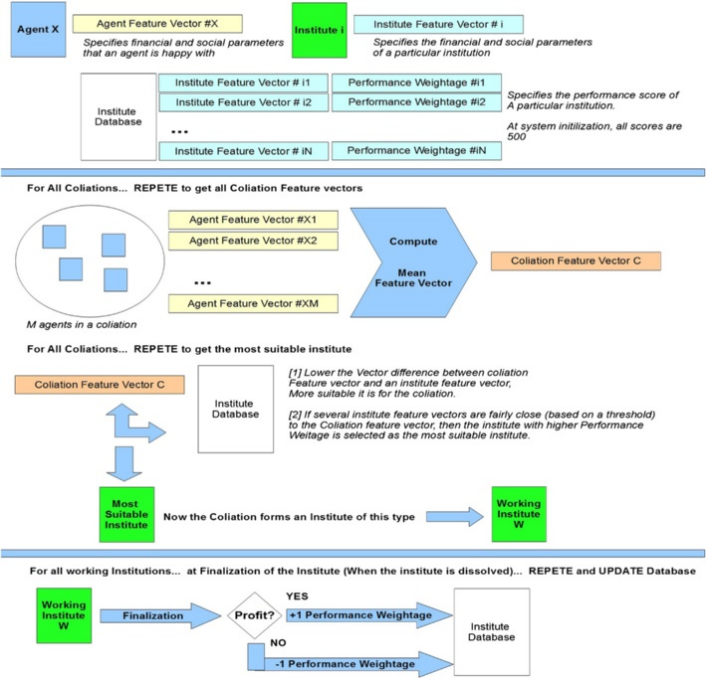
**A figure showing the table of aggregated results from the 15 experiments performed with varying controls on simulated agent ecosystems.** Key observations from these results are explained in Section 2.

ARC designs, analysis of experimental results and a scaled down version of simulation experiments on physical TurtleBOTs is presented in Figures 8 and 9. Figure 8 Part I, II show the physical TurtleBOT robots on which the scaled down versions of the experiments were performed. The Agent Relation Charts for Simulated and physical experimental setup is shown in Figure 8 Part III, IV. Due to a lower number of physical robots, the physical experiments were based on location based institution seeds. This is unlike the simulated experiments where institution seeds are assigned to agents and not their parking locations. Some run time videos of the simulation experiments and experiments on the physical TurtleBOT robots are available at (Dynamic Electronic Institutions Digital Business Ecosystem and Peer to Peer Cloud Robotics Simulation Videos 2014).

**Figure 10:**
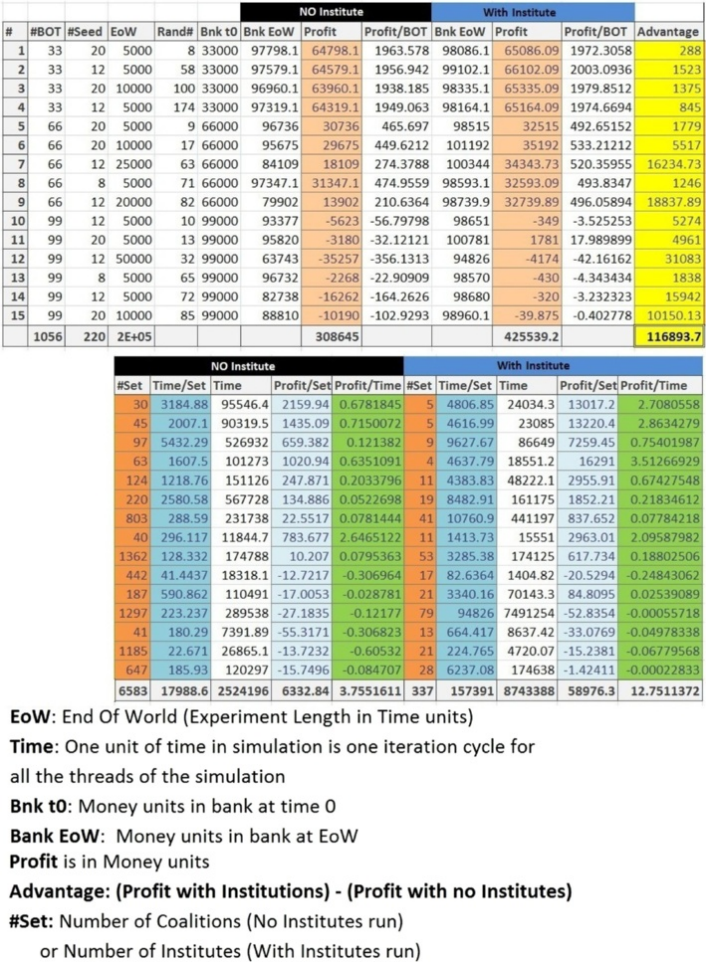
**Institution election and maintenance of institution database.**

**Institution election and Maintenance of Institution Database:** In the foundation phase of the 3F life cycle of a Dynamic Electronic Institution, member agents of a coalition negotiate amongst themselves to choose a type of institution. The mechanism can be implemented in many ways of which Case Based Reasoning and Meta-Institute approach are most common (Muntaner-Perich and Rosa Esteva 2007). For our experiments we have adopted a mixed approach where every Institute in the institute-base (a database of institutions) has a feature vector (a sequence of variables) that specifies the financial and social parameters of a particular institution. When an agent is defined, it has its own feature vector that specifies financial and social parameters that an agent is happy with. In the foundation (or re-foundation) phase (See Figure 4), the feature vectors of all the member agents of a collation are combined to form a mean feature vector. The mean feature vector is compared with the feature vectors of all the institutes in the institution base and the institute whose feature vector is closest to the mean feature vector is chosen for institution formations. Any institution with high performance is given additional weightage in the institution database. The feature vectors of institutions and the list of institutions in the database is thus dynamically adapting to the market values and profit trends. The mechanism is explained diagrammatically in Figure 10.

**Figure 11:**
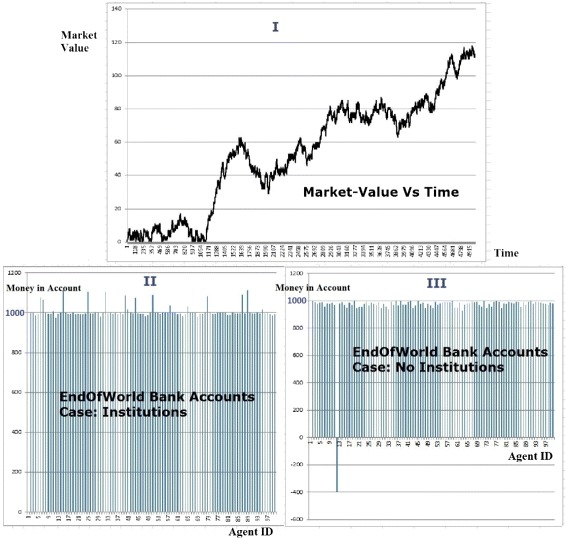
**Run time visualization of market and bank balances of agents in simulation exeriments.**

**Variability in experiment control variables:** We performed a number of different experiments on the agent colony of which 15 are presented as this case study. Figure 8 Part V shows the variation in control parameters for the 15 experiments. Figure 9 shows the control variables and results of these experiments in a tabular form. The control variables are: (1) Number of BOTs (agents) in the agent colony, (2) number of seed BOTs, (3) the time for which an experiment is allowed to run and (3) variation in market values (Figure 11 Part V shows one of the 15 different market variation patterns used for the experiments). Figure 8 Part VI to XI and Figure 9 are graphical and tabular representations of outputs of these experiments.

**Run time Visualization of Market and Bank balances of agents in simulation exeriments:** Figure 11 Part I shows sample graph of market values over a period of 5000 event steps. For every simulation run a unique random market pattern is generated that influences BOT behaviour. A randomiser seed is used to regenerate a particular market pattern. The results of running the experiment without institution formation are matched to the scenario where institutions can be formed. The profits of all BOTs for the market trend shown in Part I can be inspected at the end of the experiment as shown in Part II and III. The key observations from these experiments are explained in Section 2.

**Key Observations:**

1. Figure 8 Part VI shows profit of the agent community is always greater when they operate with institutions. This is also visible by absence of any negative value in the *Advantage* column in Figure 9.
2. Figure 8 Part VII shows average profits of agents are always greater when they operate within institutions irrespective of the size of the agent community.
3. Figure 8 Part IX shows that the number of working groups is very high when agents do not operate with institutions. This is due to greedy nature on individual agents which force a working group to dissolve when their personal goals are not fulfilled. Institutions on the other hand are very low in number as they are dissolved when a collective decision is made.
4. Another observation from Figure 8 Part IX is that the number of working groups with institutions does not fluctuate a lot with changing market patterns (As institutes are less sensitive to minor fluctuations in market trend).
5. Profit per working group is mostly higher in the case of institutions (Figure 8 Part X and columns *Profit per Set* in Figure 8).
6. Figure 8 Part VIII shows that the working groups sustain for longer duration when they operate as institutions (More work hours per working group). The peak in Experiment 12 suggests that the man hours per working group go higher as experiments run for longer duration (Experiment 12 is the longest experiment).
7. With a few exceptions, an institution based ecosystem gives more profit per work hour (Figure 8 Part XI).

## Conclusion and future direction

In this article we presented Dynamic Electronic Institutions (DEI) implementations in HTM5 meta-model for agent oriented development of cloud robotic systems. Digital Business Ecosystem (DBE) is one application domain of dynamic electronic institutions in cloud robotic colonies. HTM5 meta-model is designed for including distributed artificial intelligence designs on cloud robotic ecosystems. Peer to peer trade based on relationships between agents, representing heterogeneous cloud entities in the cloud using agents, an OMG-MDA based three layered design and its domain specificity makes HTM5 an ideal methodology for development of agent oriented cloud robotic systems. The case study, examples and discussions presented in the current article gives sufficient evidence that HTM5 is a feasible methodology for implementing complex trade and institution logics on a cloud robotic system. The complete HTM5 model, a domain specific language supporting HTM5 and a case study specific for peer to peer trade variability in HTM5 is currently submitted to well-known journals. The next step in this direction is to test the methodology in real life industrial projects and to improve the model by industrial feedback. A detailed user guide and a graphical design interface for HTM5 based designing is also currently under development.
